# Postoperative short-term mortality between insulin-treated and non-insulin-treated patients with diabetes after non-cardiac surgery: a systematic review and meta-analysis

**DOI:** 10.3389/fmed.2023.1142490

**Published:** 2023-05-02

**Authors:** Jie Jiang, Shuo Wang, Rao Sun, Yilin Zhao, Zhiqiang Zhou, Jiangjiang Bi, Ailin Luo, Shiyong Li

**Affiliations:** Department of Anesthesiology, Hubei Key Laboratory of Geriatric Anesthesia and Perioperative Brain Health, Wuhan Clinical Research Center for Geriatric Anesthesia, Tongji Hospital, Tongji Medical College, Huazhong University of Science and Technology, Wuhan, China

**Keywords:** postoperative mortality, diabetes mellitus, hypoglycemic regimens, non-cardiac surgery, insulin

## Abstract

**Background:**

Diabetes mellitus is an independent risk factor for postoperative complications. It has been reported that insulin-treated diabetes is associated with increased postoperative mortality compared to non-insulin-treated diabetes after cardiac surgery; however, it is unclear whether this finding is applicable to non-cardiac surgery.

**Objective:**

We aimed to assess the effects of insulin-treated and non-insulin-treated diabetes on short-term mortality after non-cardiac surgery.

**Methods:**

Our study was a systematic review and meta-analysis of observational studies. PubMed, CENTRAL, EMBASE, and ISI Web of Science databases were searched from inception to February 22, 2021. Cohort or case-control studies that provided information on postoperative short-term mortality in insulin-treated diabetic and non-insulin-treated diabetic patients were included. We pooled the data with a random-effects model. The Grading of Recommendations, Assessment, Development, and Evaluation system was used to rate the quality of evidence.

**Results:**

Twenty-two cohort studies involving 208,214 participants were included. Our study suggested that insulin-treated diabetic patients was associated with a higher risk of 30-day mortality than non-insulin-treated diabetic patients [19 studies with 197,704 patients, risk ratio (RR) 1.305; 95% confidence interval (CI), 1.127 to 1.511; *p* < 0.001]. The studies were rated as very low quality. The new pooled result only slightly changed after seven simulated missing studies were added using the trim-and-fill method (RR, 1.260; 95% CI, 1.076–1.476; *p* = 0.004). Our results also showed no significant difference between insulin-treated diabetes and non-insulin-treated diabetes regarding in-hospital mortality (two studies with 9,032 patients, RR, 0.970; 95% CI, 0.584–1.611; *p* = 0.905).

**Conclusion:**

Very-low-quality evidence suggests that insulin-treated diabetes was associated with increased 30-day mortality after non-cardiac surgery. However, this finding is non-definitive because of the influence of confounding factors.

**Systematic review registration:**

https://www.crd.york.ac.uk/prospero/display_record.php?ID=CRD42021246752, identifier: CRD42021246752.

## 1. Introduction

Diabetes comprises a group of metabolic disorders that is characterized by hyperglycemia, and it is a rapidly growing health problem worldwide, affecting the quality of life; diabetes had the ninth highest global mortality rate in 2010 (1–3). The International Diabetes Federation estimated that the prevalence of diabetes among adult women and men was 8.4 and 8.9%, respectively, in 2017. It is estimated the prevalence of diabetes in men and women would have increased to 9.9% by 2045 (4). The proportion of patients with diabetes who were undergoing cardiac surgery and non-cardiac surgery increased from 12.3% in 1995 to 21.2% in 2009 (5), and from 20.3% in 2004 to 25.4% in 2013 (6), respectively.

Compared to patients without diabetes, those with diabetes have a higher risk of postoperative complications, including postoperative pneumonia, wound complications, delayed wound healing, unplanned readmission, unplanned reoperation, and extended length of hospital stay (7–14). Diabetes can be divided into insulin-treated diabetes and non-insulin-treated diabetes according to the treatment regimen (15). Insulin-treated diabetes is not necessarily type 1 diabetes; it may also be type 2 diabetes that cannot be controlled with oral hypoglycemic drugs (16). Therefore, insulin-treated diabetes may be an indicator of the degree of progression of type 2 diabetes.

Meta-studies have shown that patients with insulin-treated diabetes have a higher short-term postoperative mortality risk compared to those with non-insulin-treated diabetes during cardiac surgery (17, 18). However, no such meta-analysis has been performed for non-cardiac surgery. According to a report, diabetes treated with insulin alone was an independent risk factor for death 30 days after surgery compared with diabetes treated with oral hypoglycemic drugs alone (19). The Revised Cardiac Risk Index included insulin-treated diabetes as a predictor of cardiac risk after non-cardiac surgery (20). Notably, although studies have reported higher short-term postoperative mortality in insulin-treated diabetes than in non-insulin-treated diabetes, there was no correlation between insulin-treated diabetes and postoperative mortality in multivariate logistic regression analysis (21–23). Another study also showed that in elderly patients with coronary artery disease or heart failure, insulin exposure 3 months before surgery was not associated with 30-day mortality after non-cardiac surgery (24). One study held the opposite view (25); among patients with coronary artery disease, diabetes treated with oral hypoglycemic agents was associated with a higher 2-year all-cause mortality than diabetes treated with insulin after non-cardiac surgery (25). To date, there is disagreement regarding postoperative mortality between insulin-treated diabetes and non-insulin-treated diabetes in non-cardiac surgery. Insulin-treated diabetes has been associated with more diabetes-related comorbidities and coexisting medical conditions than non-insulin-treated diabetes, and diabetes-related comorbidities and coexisting medical conditions were independent risk factors for death 30 days after non-cardiac surgery (19, 22, 26).

Therefore, we hypothesized that in non-cardiac surgery, insulin-treated diabetes would not be associated with postoperative mortality compared with non-insulin-treated diabetes after controlling for confounding factors. Patients with insulin-treated diabetes and those with non-insulin-treated diabetes were grouped according to long-term hypoglycemic regimens before admission. Considering that the hypoglycemic regimens of patients with diabetes may change after surgery, we determined the outcome as short-term postoperative mortality, including 30-day mortality and in-hospital mortality. In summary, we performed a meta-analysis of observational studies that assessed the effect of insulin-treated diabetes and non-insulin-treated diabetes on short-term mortality after non-cardiac surgery.

## 2. Methods

This study was conducted according to the Meta-analyses of Observational Studies in Epidemiology (MOOSE) (27). This study was registered with PROSPERO (CRD42021246752).

### 2.1. Data sources and search strategy

We comprehensively searched PubMed, EMBASE, CENTRAL (Cochrane Library), and Web of Science databases from inception to February 22, 2021. The retrieval strategies for each database are described in Supplementary Digital Content 1 (see Text document, Supplementary Digital Content 1, which demonstrates search strategies). The reference lists of relevant reviews were also identified.

### 2.2. Inclusion and exclusion criteria

We included studies that met each of the following criteria: (1) cohort or case-control studies; (2) inclusion of patients with diabetes who were undergoing non-cardiac surgery; (3) availability of information on 30-day mortality or in-hospital mortality regarding insulin-treated diabetes and non-insulin-treated diabetes; (4) insulin-treated diabetes and non-insulin-treated diabetes grouped according to long-term hypoglycemic regimens before admission; (5) insulin-treated diabetes referred to patients with diabetes who were receiving long-term insulin treatment before admission, including a combination of insulin and non-insulin therapy; and (6) non-insulin-treated diabetes referred to patients with diabetes who were receiving long-term non-insulin treatment before admission, excluding those who received only diet or lifestyle modifications.

We excluded studies that met any of the following criteria: (1) patients were not grouped according to pre-admission diabetes treatment regimens; (2) after contacting the authors three times, the relative risk (RR) of death and its 95% confidence interval (CI) or the odds ratio (OR) and its 95% CI or the number of deaths were not available; and (3) duplicate publications, comprising duplicate publications in different languages, duplicate publications of the same data in the same database, and articles published using the same data in the same research. Duplicate publications in different languages were translated into English using online translation software and were included as one study. For articles that collected the same data from the same database and led to repeated publications, priority was given to studies that had controlled for confounding factors. If the effect size adjusted for confounding factors was unavailable, then the latest published study was selected. For articles published using the same data in the same study, all articles were comprehensively analyzed and included as one study.

There were no language restrictions on search strategies. Non-English articles were translated into English using an online translation software.

### 2.3. Study selection

Two reviewers (RS and JJ) independently screened the titles and abstracts after using document management software to remove duplicate articles. Full-text analyses of studies that met the inclusion criteria were conducted. Finally, the included studies were determined based on the inclusion and exclusion criteria. Any differences were resolved through discussion.

### 2.4. Data extraction

Regarding the studies to be included, RS and JJ extracted the following data from the studies: the first author, publication year, study type, surgery type, demographic information of patients with insulin-treated diabetes and non-insulin-treated diabetes, RR or OR of postoperative mortality, and adjusted factors. Any differences were resolved through discussion. We extracted the death outcomes of different surgical types reported in a study.

### 2.5. Quality assessment

The Newcastle-Ottawa Scale (NOS) was used to evaluate the quality of the studies (28). A study with a score of ≥7 points was considered high quality (29, 30). In the comparability score criteria, complications and comorbidities of patients with diabetes at admission were considered the most important confounding factors. Age and American Society of Anesthesiologists (ASA) scores were considered the second most important confounding factors.

### 2.6. Data analyses

In the present study, 30-day mortality was the primary outcome, and hospital mortality was the secondary outcome. We chose the adjusted RR or OR of death to pool the data. Since the 30-day and hospital mortality after non-cardiac surgery are very low, the OR can be considered an approximate estimate of RR. Therefore, for studies in which the adjusted RR was unavailable, the adjusted OR was extracted. For studies in which neither the adjusted RR nor OR was available, we directly extracted or calculated the crude RR or OR to pool data. Although one study reported the outcome of interest, the number of deaths in both cohorts was zero. Since the RR or OR of this study could not be calculated, the data of this study was not pooled with the data of other studies. However, clinical heterogeneity was unavoidable. Therefore, a random-effects model using the DerSimonian-Laird method was used to pool the data.

A standard chi-squared test with a significance level of α = 0.1 and the *I*^2^ statistic were used to assess the magnitude of heterogeneity. *P* ≤ 0.1 or *I*^2^ ≥ 50% was considered to indicate significant heterogeneity. The source of heterogeneity was explored using subgroup and sensitivity analyses. We conducted subgroup analysis by age, surgery type, RR/OR type, NOS comparability score, and mortality type. Sensitivity analysis was performed by restricting the meta-analysis to studies with a NOS score of ≥7 points and studies that controlled for complications and comorbidities of patients with diabetes based on the design or analysis. We excluded studies one by one to assess the impact of a single study. We used the funnel plot, Begg's test, and Egger's test to evaluate publication bias. If publication bias seemed present, we used the trim-and-fill method to describe its potential impact (31). All statistical analyses were performed with STATA version 15.0. The Grading of Recommendations Assessment, Development and Evaluation (GRADE) approach was used to rate the level of evidence (32).

## 3. Results

**Figure 1:**
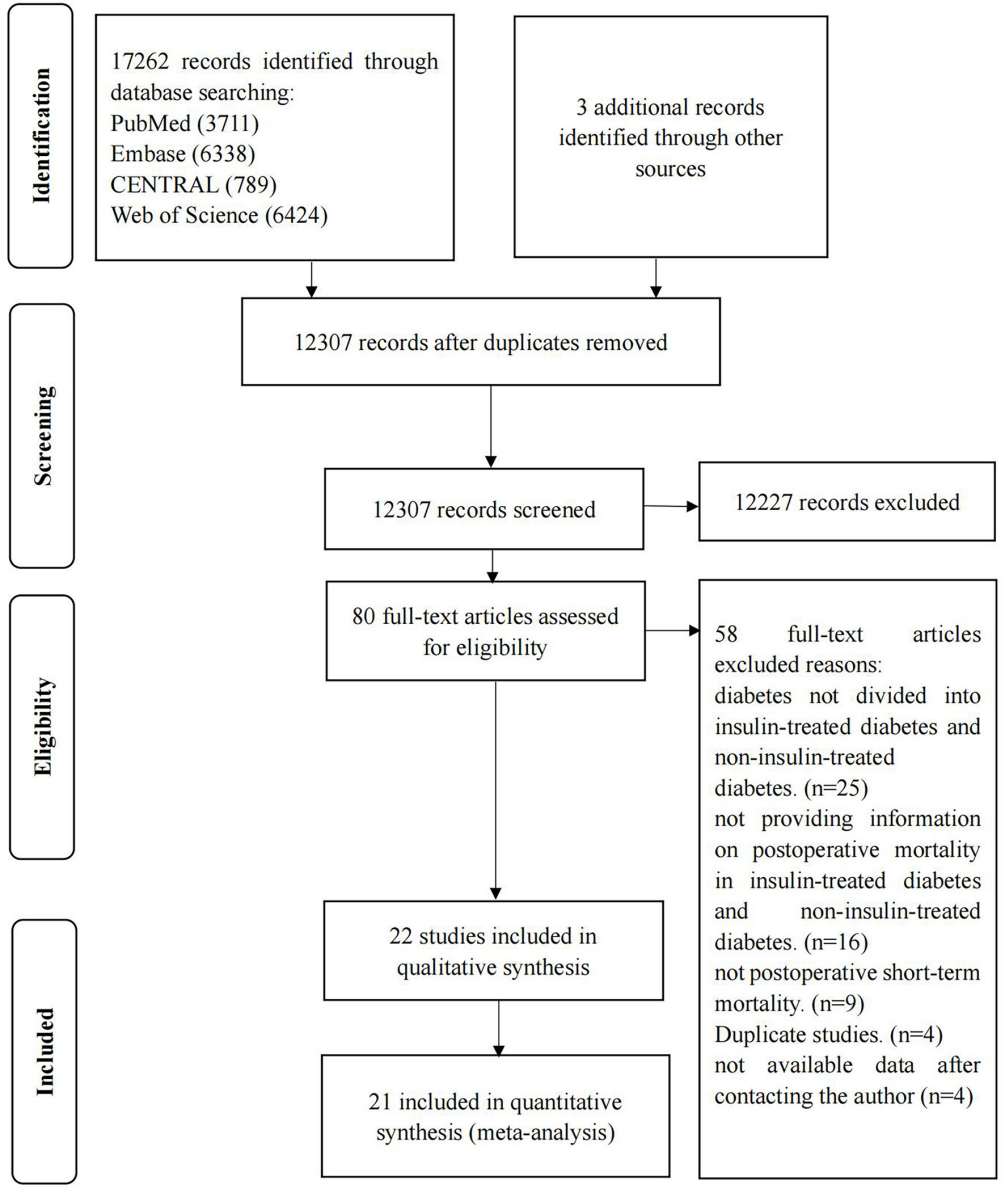
Study selection.

A total of 17,265 studies were retrieved, and 22 studies were eventually included (15, 16, 19, 22, 23, 33–49). Details of the screening process are shown in Figure 1. The excluded articles through full-text evaluation and the reasons for exclusion can be found in Supplemental Digitary Content 2 (see Text document, Supplementary Digital Content 2, which demonstrates excluded studies).

### 3.1. Characteristics of the included studies

Twenty-two studies were included, involving 208,214 patients with diabetes, of which 70,806 were insulin-treated and 137,408 were non-insulin-treated. The average age of participants in 15 studies was over 60 years (15, 19, 22, 33–35, 38–41, 43–46, 49), while the average age of participants in four studies was <60 years (37, 42, 47, 48). We were unable to determine the average age in three studies (16, 23, 36). The type of surgery in one study was breast reconstruction surgery; therefore, only female patients were included (37). Another study was based on the Department of Veterans Affairs Patient Treatment File database; therefore, 99% of the study population was male (33). In the remaining studies, the proportion of men ranged from 29.94 to 68.6%. Two studies in the study population only included patients with type 2 diabetes (19, 35). Detailed characteristics of the study are presented in Table 1.

**Table 1:** Characteristics of the included trials.

**References**	**Number ofITDM**	**Number ofNITDM**	**Total**	**Age ≥60 years**	**Male (%)**	**RR**	**Lower limitof 95% CI**	**Upper limitof 95% CI**	**RR type**	**Study type**	**Surgery type**	**Data source**	**Year of datacollection**	**Country**	**ITDMdefinition**	**NITDMdefinition**	**Types ofdiabetes**	**Mortality type**	**Adjusted factor in the analysis**
Axelrod et al. (33)	3,306	4,565	7,871	≥60	99	1.16	0.85	1.57	Crude OR	Retrospective cohort	Major vascular surgery	Department of veterans affairs patient treatment file database	1997–1999	United States	Treated with insulin	Treated with oral hypoglycemic agents or diet	Both	Hospital mortality	None
Bolliger et al. (19)	189	171	360	≥60	67.22	2.94	0.977	8.846	Crude RR	Prospective cohort	Major non-cardiac surgery	A Swiss university hospital and a Swiss tertiary-care center	2005–2009	Switzerland	Treated with insulin, including a combination of insulin and oral hypoglycaemics	Oral hypoglycaemics alone	Type 2	30-day mortality	None
Wallaert et al. (34)	620	541	1,161	≥60	67.13	0.667	0.327	1.361	Crude RR	Retrospective cohort	Lower-extremity bypass surgery	Vascular Study Group of New England	2003–2010	United States	Reliance on insulin administration at baseline to control diabetes	Patients who were diabetic, but did not rely on insulin	Both	Hospital mortality	None
Bakker et al. (35)	87	242	329	≥60	68.6	3.709	0.847	16.24	Crude RR	Retrospective cohort	Vascular surgery	Department of Vascular Surgery of the Erasmus university Medical Center	2002–2011	The Netherlands.	Treated with insulin	Treated without insulin	Type 2	30-day mortality	None
Serio et al. (36)^a^	15,050	20,747	35,797	NA	NA					Retrospective cohort					Insulin dependent	Dependent on oral hypoglycaemics	Both	30-day mortality	
Serio (vascular surgery)^a^	NA	NA	NA	NA	NA	1.06	0.83	1.35	Adjusted OR		Vascular surgery	MSQC	2007–2011	United States					Anesthesia technique, male gender, ventilator dependent, COPD, cardiac risk factors, on dialysis, steroid use, 10% weight loss, sepsis, emergent, ASA class, wound classification
Serio (general surgery)^a^	NA	NA	NA	NA	NA	1.18	1	1.39	Adjusted OR		General surgery	MSQC	2007–2011	United States					Anesthesia technique, male gender, ventilator dependent, COPD, cardiac risk factors, on dialysis, Steroid use, 10% weight loss, sepsis, emergent, ASA class, wound classification
Qin et al. (37)^b^	377	1,101	1,478	<60	0					Retrospective cohort					Treated with insulin, including a combination of insulin and non-insulin pharmacologic methods	Noninsulin pharmacologic methods, excluding diabetes controlled by diet and/or lifestyle	Both	30-day mortality	
Qin (prosthetic breast reconstruction^b^	283	815	1,098	<60	0	NA	NA	NA	NA		Prosthetic breast reconstruction	ACS-NSQIP	2005–2012	United States					None
Qin (autologous breast reconstruction)^b^	94	286	380	<60	0	NA	NA	NA	NA		Autologous breast reconstruction	ACS-NSQIP	2005–2012	United States					None
Golinvaux et al. (15)	787	1,650	2,437	≥60	45.78	2.935	0.935	9.219	Crude RR	Retrospective cohort	Elective lumbar fusion	ACS-NSQIP	2005–2012	United States	Treated with insulin, including a combination of insulin and non-insulin pharmacologic methods	Noninsulin pharmacologic methods, excluding diabetes controlled by diet and/or lifestyle	Both	30-day mortality	None
Haltmeier et al. (38)	2,280	2,280	4,560	≥60	49.6	1.177	0.998	1.388	Crude RR	Retrospective cohort	Emergency abdominal surgery	ACS-NSQIP	2005–2009	United States	Treated with insulin, including a combination of insulin and non-insulin pharmacologic methods	Noninsulin pharmacologic methods, excluding diabetes controlled by diet and/or lifestyle	Both	30-day mortality	None
Qin et al. (39)^c^	2,481	5,252	7,733	≥60	38.29					Retrospective cohort					Treated with insulin, including a combination of insulin and non-insulin pharmacologic methods	Noninsulin pharmacologic methods, excluding diabetes controlled by diet and/or lifestyle	Both	30-day mortality	
Fu et al. (40)	295	691	986	≥60	45.45	4.685	0.426	51.465	Crude RR	Retrospective cohort	Total shoulder arthroplasty	ACS-NSQIP	2011–2014	United States	Treated with insulin, including a combination of insulin and non-insulin pharmacologic methods	Noninsulin pharmacologic methods, excluding diabetes controlled by diet and/or lifestyle	Both	30-day mortality	None
Qin (Open Ventral hernia repair)^c^	2,348	4,882	7,230	≥60	38.56	0.882	0.473	1.645	Crude RR		Open ventral hernia repair	ACS-NSQIP	2005–2012	United States					None
Qin (laparoscopic ventral hernia repair)^c^	133	370	503	<60	34.4	0.923	0.038	22.517	Crude RR		Laparoscopic Ventral hernia repair	ACS-NSQIP	2005–2012	United States					None
Patterson et al. (41)	91	153	244	≥60	29.94	3.363	0.309	36.566	Crude RR	Retrospective cohort	Proximal humerus fractures	ACS-NSQIP	2005–2014	United States	Treated with insulin, including a combination of insulin and non-insulin pharmacologic methods	Noninsulin pharmacologic methods, excluding diabetes controlled by diet and/or lifestyle	Both	30-day mortality	None
Phan et al. (42)	171	270	441	<60	51.25	4.727	0.194	115.369	Crude RR	Retrospective cohort	Anterior cervical discectomy and fusion	ACS-NSQIP	2005–2012	United States	Treated with insulin, including a combination of insulin and non-insulin pharmacologic methods	Noninsulin pharmacologic methods, excluding diabetes controlled by diet and/or lifestyle	Both	30-day mortality	None
Webb et al. (43)	4,881	15,367	20,248	≥60	40.85	1.296	0.538	3.124	Crude RR	Retrospective cohort	Total knee arthroplasty	ACS-NSQIP	2005–2014	United States	Treated with insulin, including a combination of insulin and non-insulin pharmacologic methods	Noninsulin pharmacologic methods, excluding diabetes controlled by diet and/or lifestyle	Both	30-day mortality	None
Di Capua et al. (44)	250	540	790	≥60	45.79	0.617	0.129	2.95	Crude RR	Retrospective cohort	Elective adult spinal deformity surgery	ACS-NSQIP	2010–2014	United States	Treated with insulin, including a combination of insulin and non-insulin pharmacologic methods	Noninsulin pharmacologic methods, excluding diabetes controlled by diet and/or lifestyle	Both	30-day mortality	None
Stepan et al. (45)	1,772	2,679	4,451	≥60	42.42	3.402	1.049	11.029	Crude RR	Retrospective cohort	Upper extremity surgery	ACS-NSQIP	2005–2015	United States	Treated with insulin, including a combination of insulin and non-insulin pharmacologic methods	Noninsulin pharmacologic methods, excluding diabetes controlled by diet and/or lifestyle	Both	30-day mortality	None
Pothof et al. (46)	1,919	3,118	5,037	≥60	62.16	1.937	1.154	3.252	Crude RR	Retrospective cohort	Carotid endarterectomy	ACS-NSQIP	2011–2015	United States	Treated with insulin, including a combination of insulin and non-insulin pharmacologic methods	Noninsulin pharmacologic methods, excluding diabetes controlled by diet and/or lifestyle	Both	30-day mortality	None
Traven et al. (16)	2,484	5,332	7816	NA	56.89	2.147	0.622	7.408	Crude RR	Retrospective cohort	Shoulder arthroscopy	ACS-NSQIP	2005–2016	United States	Treated with insulin, including a combination of insulin and non-insulin pharmacologic methods	Noninsulin pharmacologic methods, excluding diabetes controlled by diet and/or lifestyle	Both	30-day mortality	None
Gu et al. (22)	975	1,890	2,865	≥60	44.86	10.752	0.501	250	Adjusted OR	Retrospective cohort	Revision total knee arthroplasty	ACS-NSQIP	2007–2016	United States	Treated with insulin, including a combination of insulin and non-insulin pharmacologic methods	Noninsulin pharmacologic methods, excluding diabetes controlled by diet and/or lifestyle	Both	30-day mortality	NA
Leonard-Murali et al. (23)^d^	29,361	60,012	89,373	NA	NA					Retrospective cohort					Requiring daily insulin therapy	Requiring therapy with a noninsulin anti-diabetic agent	Both	30-day mortality	
Leonard-Murali (laparoscopic sleeve gastrectomy)^d^	16,342	40,414	56,756	NA	NA	1.23	0.73	2.08	Adjusted OR		Laparoscopic sleeve gastrectomy	MBSAQIP	2015–2017	United States					Age, sex, race, ASA class, and BMI
Leonard-Murali (laparoscopic Roux-en-Y gastric bypass)^d^	13,019	19,598	32,617	NA	NA	1.33	0.78	2.25	Adjusted OR		Laparoscopic Roux-en-Y gastric bypass	MBSAQIP	2015–2017	United States					Age, sex, race, ASA class, and BMI
Mamidi et al. (47)	211	379	590	<60	32.39	5.377	0.22	131.419	Crude RR	Retrospective cohort	Tonsillectomy	ACS-NSQIP	2005–2018	United States	Treated with insulin, including a combination of insulin and non-insulin pharmacologic methods	Noninsulin pharmacologic methods, excluding diabetes controlled by diet and/or lifestyle	Both	30-day mortality	None
Selemon et al. (48)	334	700	1,034	<60	47.16	0.466	0.101	2.144	Crude RR	Retrospective cohort	Aseptic revision total hip arthroplasty	ACS-NSQIP	2006–2016	United States	Treated with insulin, including a combination of insulin and non-insulin pharmacologic methods	Noninsulin pharmacologic methods, excluding diabetes controlled by diet and/or lifestyle	Both	30-day mortality	None
Kebaish et al. (49)	2,885	9,728	12,613	≥60	50.96	2.698	1.066	6.828	Crude RR	Retrospective cohort	Total hip arthroplasty	ACS-NSQIP	2012–2016	United States	Treated with insulin, including a combination of insulin and non-insulin pharmacologic methods	Noninsulin pharmacologic methods, excluding diabetes controlled by diet and/or lifestyle	Both	30-day mortality	None

ITDM, insulin-treated diabetes mellitus; NITDM, non-insulin-treated diabetes mellitus; NA, not available; RR, relative risk; CI, confidence interval; OR, odds ratio; ACS-NSQIP, American College of Surgeons National Surgical Quality Improvement Program Database; MBSAQIP, Metabolic and Bariatric Surgery Accreditation and Quality Improvement Program database; MSQC, Michigan Surgical Quality Collaborative; COPD, chronic obstructive pulmonary disease; ASA, American Society of Anesthesiologists; BMI, body mass index.

a Serio et al. (36) provided mortality information for vascular surgery and general surgery, respectively.

b Qin et al. (37) provided mortality information for prosthetic breast reconstruction and autologous breast reconstruction, respectively.

c Qin et al. (38) provided mortality information for open ventral hernia repair and laparoscopic ventral hernia repair, respectively.

d Leonard-Murali et al. (23) provided mortality information for laparoscopic sleeve gastrectomy and laparoscopic Roux-en-Y gastric bypass, respectively.

The NOS scores of the studies ranged from 4 to 7 points. Most of them scored five points. Only two studies were rated as high quality (38, 47). The details of the NOS scores are shown in Table 2. Regarding the comparability score, only three studies controlled for the most important confounding factor (36, 38, 47), and only three studies controlled for the second most important confounding factor (23, 38, 47).

**Table 2:** Newcastle-Ottawa Scale (cohort studies).

**References**	**Selection**				**Comparability**		**Outcome**			**Quality**
	**Representativeness of the exposed cohort**	**Selection of the non-exposed cohort**	**Ascertainment of exposure**	**Demonstration that outcome of interest was not present at start of study**	**Comparability of cohorts on the basis of the design or analysis (the most important factor)**	**Comparability of cohorts on the basis of the design or analysis (the second factor)**	**Assessment of outcome**	**Was follow-up long enough for outcomes to occur**	**Adequacy of follow up of cohorts**	
Axelrod et al. (33)	^*^	–	^*^	–	–	–	^*^	^*^	–	4
Bolliger et al. (19)	–	^*^	^*^	^*^	–	–	^*^	^*^	^*^	6
Wallaert et al. (34)	^*^	–	^*^	–	–	–	^*^	^*^	–	4
Bakker et al. (35)	–	–	^*^	–	–	–	^*^	^*^	–	3
Serio et al. (36)	^*^	^*^	^*^	–	^*^	–	^*^	^*^	–	6
Golinvaux et al. (15)	^*^	^*^	^*^	–	–	–	^*^	^*^	–	5
Qin et al. (37)	^*^	^*^	^*^	–	–	–	^*^	^*^	–	5
Haltmeier et al. (38)	^*^	^*^	^*^	–	^*^	^*^	^*^	^*^	–	7
Qin et al. (39)	^*^	^*^	^*^	–	–	–	^*^	^*^	–	5
Fu et al. (40)	^*^	^*^	^*^	–	–	–	^*^	^*^	–	5
Patterson et al. (41)	^*^	^*^	^*^	–	–	–	^*^	^*^	–	5
Phan et al. (42)	^*^	^*^	^*^	–	–	–	^*^	^*^	–	5
Webb et al. (43)	^*^	^*^	^*^	–	–	–	^*^	^*^	–	5
Di Capua et al. (44)	^*^	^*^	^*^	–	–	–	^*^	^*^	–	5
Stepan et al. (45)	^*^	^*^	^*^	–	–	–	^*^	^*^	–	5
Pothof et al. (46)	^*^	^*^	^*^	–	–	–	^*^	^*^	–	5
Traven et al. (16)	^*^	^*^	^*^	–	–	–	^*^	^*^	–	5
Gu et al. (22)	^*^	^*^	^*^	–	–	–	^*^	^*^	–	5
Leonard-Murali et al. (23)	^*^	^*^	^*^	–	–	^*^	^*^	^*^	–	6
Mamidi et al. (47)	^*^	^*^	^*^	–	^*^	^*^	^*^	^*^	–	7
Selemon et al. (48)	^*^	^*^	^*^	–	–	–	^*^	^*^	–	5
Kebaish et al. (49)	^*^	^*^	^*^	–	–	–	^*^	^*^	^*^	6

* The scale uses a star system, with a maximum of score of 9 stars, and includes 3 categories: selection, comparability, and outcome. Asterisk represents one point.

### 3.2. Thirty-day mortality

**Figure 2:**
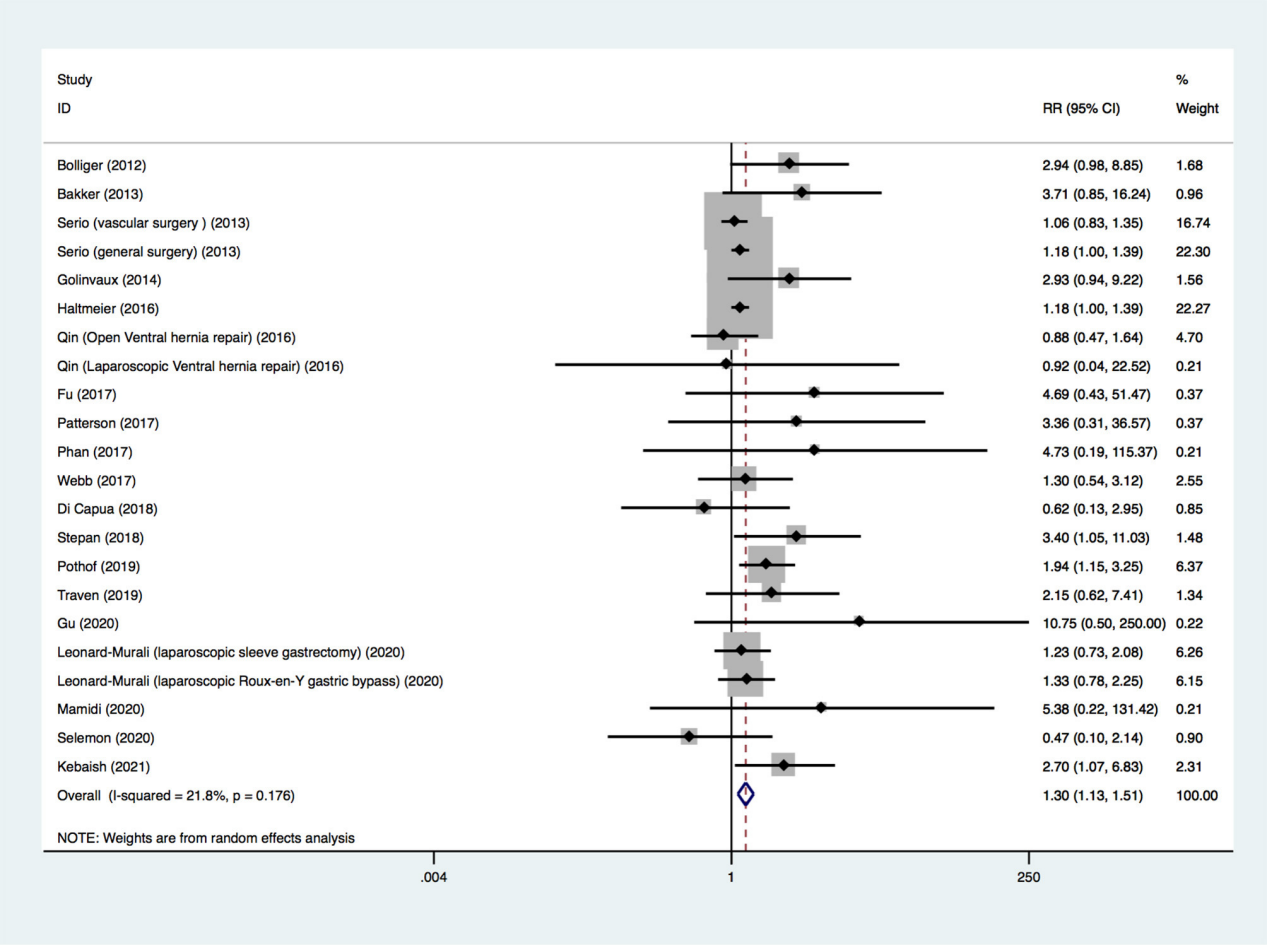
Forest plot for risk of 30-day mortality.

The 20 included studies provided information on the outcome of 30-day mortality. Because four studies provided death outcomes for different types of surgery (23, 36, 37, 39), there were 24 groups of postoperative death data. Because the death outcomes of insulin-treated diabetes and non-insulin-treated diabetes in one study were both zero events (37), the data from this study could not be combined with data from other studies. Finally, 22 sets of data (19 studies, 206,736 participants) were pooled. Among these 22 sets, five were the adjusted OR, and the remaining 17 were the crude RR. A random-effects model was used to pool all the data, and a significant difference was found (RR, 1.305; 95% CI, 1.127–1.511; *p* < 0.001; Figure 2), suggesting that insulin-treated diabetes had a higher risk of 30-day mortality compared to non-insulin-treated diabetes. The quality of evidence was rated as very low. The details of the GRADE summary of the findings are shown in Supplementary Digital Content 3 (see Table, Supplementary Digital Content 3, which demonstrates GRADE).

We conducted subgroup analyses by the age of participants, type of surgery, RR/OR type, and comparability score of NOS (Supplemental Digital Content 4). In the subgroups of aged ≥ 60 years, orthopedic surgery, general surgery, upper extremity surgery, crude RR, adjusted OR, comparability score of 0-point, 1-point and 2-point, the results showed a higher risk of 30-day mortality of insulin-treated diabetes compared to non-insulin-treated diabetes. In the subgroups of aged <60 years, vascular surgery, ventral hernia repair, bariatric surgery, emergency abdominal surgery, tonsillectomy, major non-cardiac surgery, the results showed a similar 30-day mortality between insulin-treated diabetes and non-insulin-treated diabetes.

The details of the subgroup analysis are presented in Table 3.

**Table 3:** Results of subgroup and sensitivity analyses for risk of 30-day mortality.

	**No. of studies**	**No. of data**	**No. of patients**	**Heterogeneity**	**Pooled risk ratio (95% CI)**	**Significance**
**Age of participants**						
<60 yr	4^a^	4^a^	25,68	*I*-squared = 0.0%, *P* = 0.402	0.975 (0.301, 3.162)	*p* = 0.967
≥60 yr	13^a^	13^a^	62,150	*I*-squared = 41.3%, *P*= 0.059	1.716 (1.244, 2.366)	*p* = 0.001
Unknown	3^b^, ^c^	5^b^, ^c^	132,986	*I*-squared = 0.0%, *P* = 0.769	1.164 (1.025, 1.322)	*p* = 0.019
**Type of surgery**						
Orthopedic surgery	10	10	49,474	*I*-squared = 3.7%, *P* = 0.406	1.827 (1.165, 2.865)	*p* = 0.009
Vascular surgery	3^b^	3^b^	^d^	*I*-squared = 69.3%, *P* = 0.038	1.560 (0.861, 2.827)	*p* = 0.143
Ventral hernia repair	1^a^	2^a^	7,733	*I*-squared = 0.0%, *P*= 0.978	0.883 (0.479, 1.629)	*p* = 0.691
Bariatric surgery	1^c^	2^c^	89,373	*I*-squared = 0.0%, *P* = 0.837	1.278 (0.881, 1.855)	*p* = 0.196
Emergency abdominal surgery	1	1	4,560	–	1.177 (0.998, 1.388)	*p* = 0.053
Tonsillectomy	1	1	590	–	5.377 (0.220, 131.419)	*p* = 0.302
Major non-cardiac surgery	1	1	360	–	2.940 (0.977, 8.847)	*p* = 0.055
General surgery	1^b^	1^b^	^e^	–	1.180 (1.001, 1.391)	*p* = 0.049
Upper extremity surgery	1	1	4,451	–	3.402 (1.049, 11.031)	*p* = 0.041
**RR/OR type**						
Crude RR	16^a^	17^a^	69,669	*I*-squared = 28.8%, *P* = 0.129	1.614 (1.218, 2.138)	*p* = 0.001
Adjusted OR	3	5^b^, ^c^	128,035	*I*-squared = 0.0%, *P* = 0.584	1.161 (1.021, 1.319)	*p* = 0.022
**Comparability score of NOS**						
0 point	15^a^	16^a^	67,384	*I*-squared = 13.8%, *P* = 0.296	1.802 (1.317, 2.466)	*p* < 0.001
1 point	2^b^, ^c^	4^b^, ^c^	125,170	*I*-squared = 0.0%, *P* = 0.832	1.156 (1.017, 1.314)	*p* = 0.026
2 points	2	2	5,150	*I*-squared = 0.0%, *P* = 0.352	1.182 (1.002, 1.393)	*p* = 0.047

a Qin et al. (39) provided mortality information for open ventral hernia repair and laparoscopic ventral hernia repair, respectively.

b Serio et al. (36) provided mortality information for vascular surgery and general surgery, respectively.

c Leonard-Murali et al. (23) provided mortality information for laparoscopic sleeve gastrectomy and laparoscopic Roux-en-Y gastric bypass, respectively.

d Serio et al. (36) did not provide demographic information for vascular surgery.

e Serio et al. (36) did not provide demographic information for general surgery.

We also conducted sensitivity analysis. Limiting the analysis to studies with a NOS score ≥ 7, the results were consistent with the original analysis (RR, 1.182; 95% CI, 1.002–1.393; *p* = 0.047). Limiting the analysis to the studies that controlled for complications and comorbidities of patients with diabetes based on the design or analysis, the results were consistent with the original analysis (RR, 1.157; 95% CI, 1.042–1.286; *p* = 0.006). The included studies were excluded one by one, and no single study had a noticeable influence on the total combined effect size (see Text document, Supplementary Digital Content 4, which demonstrates sensitivity analysis).

The publication bias was evaluated. The funnel plot appeared asymmetrical (see Text document, Supplementary Digital Content 5, which demonstrates funnel plot). Significant publication bias was detected using Egger's test (bias = 0.88; 95% CI, 0.31–1.44; *p* = 0.004). After using the trim-and-fill method, the new pooled effect size from the random-effects model was similar to the primary result (RR, 1.260; 95% CI, 1.076–1.476; *p* = 0.004). A new funnel plot after adding seven simulated missing studies is presented in Supplementary Digital Content 6 (see Text document, Supplementary Digital Content 6, which demonstrates new funnel plot).

### 3.3. Hospital mortality

**Figure 3:**
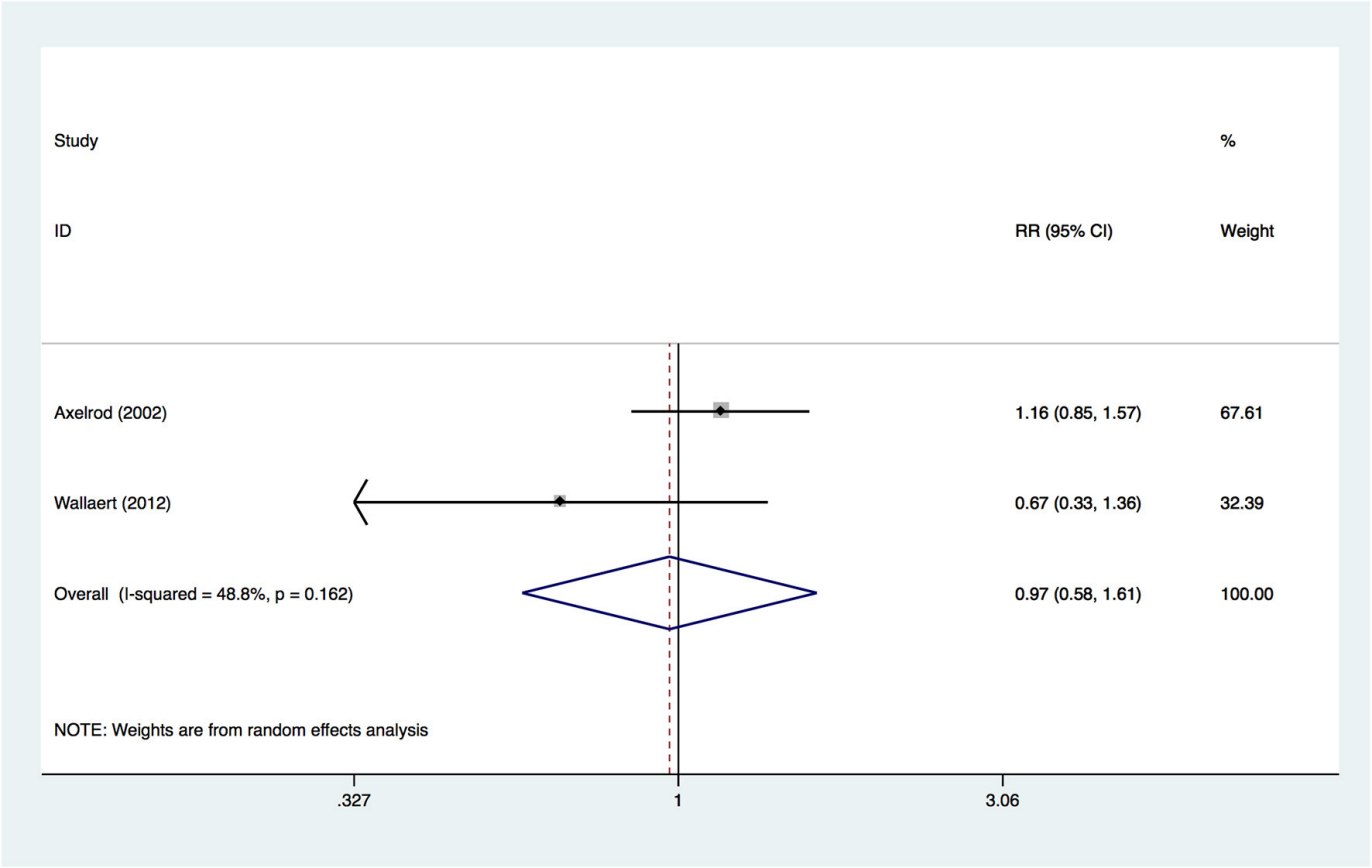
Forest plot for risk of in-hospital mortality.

Two included studies provided information on the outcome of hospital mortality. A random-effects model was used to pool the data, and no significant difference was found (RR, 0.970; 95% CI, 0.584–1.611; *p* = 0.905; Figure 3) between insulin-treated diabetes and non-insulin-treated diabetes, suggesting that the two groups had a similar hospital mortality. The quality of evidence was rated as very low. The details of the GRADE summary of the findings are shown in Supplementary Digital Content 3 (see Table, Supplementary Digital Content 3, which demonstrates GRADE).

As only two studies were included in the outcome, we did not conduct subgroup analysis, sensitivity analysis or evaluate publication bias.

## 4. Discussion

Our study demonstrated that compared with non-insulin-treated diabetes, insulin-treated diabetes was associated with a higher risk of 30-day mortality, but a similar risk of in-hospital mortality after surgery. The quality of the evidence was rated as very low.

### 4.1. Comparison with the published literature

Our findings are consistent with two previously published systematic reviews of cardiac surgery that found that insulin-treated diabetes had a significantly higher risk of short-term mortality (<1 year) after percutaneous coronary intervention (18) and a significantly higher risk of short-term mortality ( ≤ 30 days) after coronary artery bypass surgery compared with non-insulin-treated diabetes (17).

Although all the included studies provided postoperative death outcomes for insulin-treated diabetes and non-insulin-treated diabetes, only seven studies specifically compared postoperative mortality outcomes between insulin-treated and non-insulin-treated diabetes (19, 22, 23, 33, 36, 38, 47). Only one study found that insulin-treated diabetes was associated with increased postoperative mortality compared to non-insulin-treated diabetes (19). However, in this study, the relationship only held for insulin-only diabetes, and did not include diabetes treated both with and without insulin.

It has also been reported that neither diabetes nor insulin exposure is an independent risk factor for death at 30 days after non-cardiac surgery in 65-year-old patients with coronary artery disease or heart failure (24). Among studies with longer follow-up times after non-cardiac surgery, some studies found insulin-treated diabetes to be an independent risk factor for postoperative death compared with non-insulin-treated diabetes (50, 51), while one study found no association (52). One study found that oral hypoglycemic therapy, but not insulin therapy, was associated with non-cardiac mortality at 2 years in patients at coronary risk (25).

### 4.2. Strengths

Our study had several strengths. First, to our knowledge, it is the first systematic review comparing short-term postoperative mortality between insulin-treated diabetes and non-insulin-treated diabetes in non-cardiac surgery. Second, we screened 17,265 articles and finally included 22 studies that comprised 208,214 patients with diabetes; thus, our study comprised a notably large number of patients. Third, no significant heterogeneity was found in our study (*I*^2^= 22.5%, *p* = 0.159). Although there was significant publication bias, the new pooled result was only slightly changed after adding the simulated missing study using the trim-and-fill method, suggesting that the effect of publication bias on the result might be small. Fourth, of the studies we included, only two included type 2 diabetes (19, 35). The remaining 21 studies did not clarify the type of diabetes. The patients were grouped based on prehospital hypoglycemic regimens. This shows that our conclusions are practical.

### 4.3. Implications for clinicians, policy, and research

Our study showed that insulin-treated diabetes and non-insulin-treated diabetes had a similar risk of in-hospital mortality after surgery. However, only two studies were included in the outcome, thus, the findings may be not reliable. Our study also showed that in non-cardiac surgery, the 30-day mortality of insulin-treated patients with diabetes was higher than that of non-insulin-treated diabetes patients. This suggests that prehospital hypoglycemic regimens might be one of the bases for the preoperative risk stratification of patients with diabetes. However, it must be noted that because of the generally low quality of the originally included studies, this conclusion may be unreliable. In particular, only three studies controlled the bias of comorbidities and complications of patients with diabetes in the study design or data analysis. Only one of the originally included studies found that type 2 diabetes treated with insulin alone was an independent risk factor for short-term postoperative death compared with diabetes treated with oral hypoglycemic agents alone. However, the results of the study might be explained by its inclusion of more patients with a history of myocardial infarction, more patients with a history of coronary artery bypass graft, more patients with a history of heart failure, and a higher ASA grade in the insulin-only group. Surprisingly, insulin-treated diabetes was still associated with increased postoperative mortality but with a reduced effect size in our sensitivity analyses that included only studies that controlled for comorbidities and complications and only high-quality studies. This suggests on the one hand that confounders caused an overestimation of the effect size, and on the other hand, long-term prehospital hypoglycemic regimens might indeed influence short-term postoperative mortality. This might be related to the long-term cardiovascular protective effects of oral hypoglycemic agents (53–55).

In published models predicting postoperative cardiac complications for non-cardiac surgery, the Revised Cardiac Risk Index included preoperative treatment with insulin as a risk factor (20), while the Geriatric-Sensitive Perioperative Cardiac Risk Index included diabetes as a risk factor (56). Although it is still not clear whether the hypoglycemic regimen or the severity of the patient's condition leads to increased short-term postoperative mortality, our current results still tended to stratify diabetes patients according to prehospital hypoglycemic regimens and assign insulin-treated diabetes with a higher risk level.

Sensitivity and subgroup analyses with adjusted effect sizes showed that insulin-treated diabetes was an independent risk factor. However, in the subgroup analysis of surgical types, only the subgroup of orthopedic surgery and vascular surgery could be further analyzed, while the number of studies on other surgical types was too small to be further analyzed. In the vascular surgery subgroup, insulin-treated diabetes was not associated with increased 30-day mortality. This suggests that there is a need to further distinguish the risks of long-term hypoglycemic regimens between different surgical types. There was no association between insulin-treated diabetes and 30-day mortality in the subgroup of age <60 years. This indicates that our results may not be applicable to all populations. It is also possible that the severity of diabetes might be similar in patients undergoing vascular surgery or in patients aged <60 years. However, our current research was unable to answer this question; thus, well-designed and high-quality research are required to answer these questions in the future.

## 5. Limitation

The most notable limitation of our study is that the quality of most of the originally included studies was not high. Of the 22 studies, only two were rated as high-quality studies. Diabetes-related complications and comorbidities were independent risk factors for non-cardiac postoperative 30-day mortality (26). Age and ASA grade were also listed as risk factors for major adverse cardiovascular events and mortality within 30 days after surgery (56–58). Therefore, we regarded diabetes-related complications and comorbidities as the most important confounding factors, while age and ASA grade were the second most important confounding factors. Of the 22 studies included, only three controlled for diabetes-related complications and comorbidities, and only three controlled for age and ASA classification. The evidence quality rating was very low. This showed that the results obtained are uncertain.

In the subgroup analysis, the results of the vascular surgery and age <60 subgroups were not consistent with the original analysis. Thus, our study conclusions may not be applicable to all surgery types and populations.

## 6. Conclusion

Our study showed that insulin-treated diabetes was associated with an increased risk of 30-day mortality after non-cardiac surgery compared with non-insulin-treated diabetes. The quality of the evidence was rated as very low. As it is difficult to rule out the influence of confounding factors, we believe that the results obtained are not reliable. Well-designed, high-quality studies that controls for important confounding factors is needed to investigate the impact of prehospital hypoglycemic regimens on short-term mortality after non-cardiac surgery.

## Data availability statement

The original contributions presented in the study are included in the article/Supplementary material, further inquiries can be directed to the corresponding author.

## Author contributions

JJ and SW: methodology and writing—original draft preparation. RS and SL: data curation and writing—review and editing. YZ and ZZ: methodology and writing—editing. JB: data curation and funding acquisition. AL: funding acquisition and supervision. All authors contributed to the article and approved the submitted version.
