# Supplementary material for: Postoperative short-term mortality between insulin-treated and non-insulin-treated patients with diabetes after non-cardiac surgery: a systematic review and meta-analysis
Source: Front Med (Lausanne). 2023 May 2;10:1142490. doi: 10.3389/fmed.2023.1142490 (PMC10185903; doi:10.3389/fmed.2023.1142490)
Supplement: Supplementary file 1 [file Data_Sheet_1.zip › Supplemental Digital Content 1.docx]

Supplemental Digital Content 1

Postoperative short-term mortality between insulin-treated and non-insulin-treated patients with diabetes after non-cardiac surgery: a systematic review and meta-analysis

##

## Search strategies

Pubmed

#1 (diabetes[Title/Abstract]) OR (diabetic[Title/Abstract])

#2 (((((((((Specialties, Surgical[MeSH Terms]) OR (Surgical Procedures, Operative[MeSH Terms])) OR (Postoperative Complications[MeSH Terms])) OR (surger*[Title/Abstract])) OR (surgical[Title/Abstract])) OR (operat*[Title/Abstract])) OR (grafting[Title/Abstract])) OR (transplant*[Title/Abstract])) OR (postoperative[Title/Abstract])) OR (procedure*[Title/Abstract])

#3 insulin[Title/Abstract]

#4 ((((((oral[Title/Abstract]) OR (non-insulin[Title/Abstract])) OR (insulin-requiring[Title/Abstract])) OR ("requiring insulin"[Title/Abstract])) OR (insulin-treated[Title/Abstract])) OR (IDDM[Title/Abstract])) OR ("not treated with insulin"[Title/Abstract])#6 (control [Title/Abstract]) AND (blood glucose [Title/Abstract])

#5 #1 AND #2 AND #3 AND #4

EMBASE

#1 diabetes:ab,ti OR diabetic:ab,ti

#2 'specialties, surgical'/exp OR 'surgical procedures, operative'/exp OR 'postoperative complications'/exp OR surger*:ab,ti OR surgical:ab,ti OR operat*:ab,ti OR grafting:ab,ti OR transplant*:ab,ti OR postoperative:ab,ti OR procedure*:ab,ti

#3 insulin:ab,ti

#4 oral:ab,ti OR 'non insulin':ab,ti OR 'insulin requiring':ab,ti OR 'requiring insulin':ab,ti OR 'insulin treated':ab,ti OR iddm:ab,ti OR 'not treated with insulin':ab,ti

#5 #1 AND #2 AND #3 AND #4

CENTRAL (by The Cochrane Library)

#1 (diabetes):ti,ab,kw OR (diabetic):ti,ab,kw

#2 MeSH descriptor: [Specialties, Surgical] explode all trees

#3 MeSH descriptor: [Surgical Procedures, Operative] explode all trees

#4 MeSH descriptor: [Postoperative Complications] explode all trees

#5 (surger*):ti,ab,kw OR (surgical):ti,ab,kw OR (operat*):ti,ab,kw OR (grafting):ti,ab,kw OR (transplant*):ti,ab,kw

#6 (postoperative):ti,ab,kw OR (procedure*):ti,ab,kw

#7 (insulin):ti,ab,kw

#8 (oral):ti,ab,kw OR (non-insulin):ti,ab,kw OR (insulin-requiring):ti,ab,kw OR (“requiring insulin”):ti,ab,kw OR (insulin-treated):ti,ab,kw

#9 (IDDM):ti,ab,kw OR (“not treated with insulin”):ti,ab,kw

#10 #1 AND (#2 OR #3 OR #4 OR #5 OR #6) AND #7 AND (#8 OR #9)

Web of Science

#1 AB= (diabetes OR diabetic)

#2 AB= (surger* OR surgical OR postoperative)

#3 AB=(insulin)

#4 AB=(oral OR non-insulin)

#5 #4 AND #3 AND #2 AND #1
