# Supplementary material for: Postoperative short-term mortality between insulin-treated and non-insulin-treated patients with diabetes after non-cardiac surgery: a systematic review and meta-analysis
Source: Front Med (Lausanne). 2023 May 2;10:1142490. doi: 10.3389/fmed.2023.1142490 (PMC10185903; doi:10.3389/fmed.2023.1142490)
Supplement: Supplementary file 1 [file Data_Sheet_1.zip › Supplemental Digital Content 2.docx]

Supplemental Digital Content 2

Postoperative short-term mortality between insulin-treated and non-insulin-treated patients with diabetes after non-cardiac surgery: a systematic review and meta-analysis

## Excluded studies

| Reasons for exclusion | Studies |
| --- | --- |
| Diabetes not divided into insulin-treated diabetes and non-insulin-treated diabetes. (n=25) | ^1-25^ |
| Not providing information on postoperative mortality in insulin-treated diabetes and non-insulin-treated diabetes. (n=16) | ^26-41^ |
| Not postoperative short-term mortality. (n=9) | ^42-50^ |
| Duplicate studies. (n=4) | ^51-54^ |
| Not available data after contacting the author (n=4) | ^54-58^ |

1. Vannini P, Ciavarella A, Olmi R et al. Diabetes as pro-infective risk factor in total hip replacement. *Acta diabetologica latina* 1984; **21**:275-280.

2. Boucek P, Pokorna E, Saudek F, Vitko S, Lanska V. Kidney transplantation in Type 2 diabetes mellitus: A case-control comparison with non-diabetic subjects. *Diabetes Research and Clinical Practice* 2000; **50**:S262-S262.

3. John PR, Thuluvath PJ. Outcome of liver transplantation in patients with diabetes mellitus: a case-control study. *Hepatology* 2001; **34**:889-895.

4. Chan PKH, Brenkel IJ, Aderinto J. The outcome of total hip arthroplasty in diabetes mellitus. *British Journal of Diabetes and Vascular Disease* 2005; **5**:146-149.

5. Schiel R, Heinrich S, Steiner T, Ott U, Stein G. Long-term prognosis of patients after kidney transplantation: a comparison of those with or without diabetes mellitus. *Nephrology Dialysis Transplantation* 2005; **20**:611-617.

6. Bradbury RA, Shirkhedkar D, Glanville AR, Campbell LV. Prior diabetes mellitus is associated with increased morbidity in cystic fibrosis patients undergoing bilateral lung transplantation: An 'orphan' area? A retrospective case-control study. *Internal Medicine Journal* 2009; **39**:384-388.

7. Ata A, Valerian BT, Lee EC, Bestle SL, Elmendorf SL, Stain SC. The effect of diabetes mellitus on surgical site infections after colorectal and noncolorectal general surgical operations. *American Surgeon* 2010; **76**:697-702.

8. Teber D, Sofikerim M, Ates M et al. Is Type 2 Diabetes Mellitus a Predictive Factor for Incontinence After Laparoscopic Radical Prostatectomy? A Matched Pair and Multivariate Analysis. *Journal of Urology* 2010; **183**:1087-1091.

9. Vavallo A, Lucarelli G, Tedeschi M et al. Diabetes Mellitus Is a Risk Factor for Recurrence and Mortality in Patients with Renal Cell Carcinoma. *European Urology Supplements* 2011; **10**:139-140.

10. Antonelli A, Arrighi N, Corti S et al. Pre-existing type-2 diabetes is not an adverse prognostic factor in patients with renal cell carcinoma: A single-center retrospective study. *Urologic Oncology: Seminars and Original Investigations* 2013; **31**:1310-1315.

11. Ekström W, Al-Ani AN, Sääf M, Cederholm T, Ponzer S, Hedström M. Health related quality of life, reoperation rate and function in patients with diabetes mellitus and hip fracture--a 2 year follow-up study. *Injury* 2013; **44**:769-775.

12. Hoefner T, Zeier M, Hatiboglu G et al. The impact of type 2 diabetes on the outcome of localized renal cell carcinoma. *World Journal of Urology* 2014; **32**:1537-1542.

13. Wei Z-W, Li J-L, Wu Y et al. Impact of Pre-existing Type-2 Diabetes on Patient Outcomes After Radical Resection for Gastric Cancer: A Retrospective Cohort Study. *Digestive Diseases and Sciences* 2014; **59**:1017-1024.

14. Hajjij A, Mace JC, Soler ZM, Smith TL, Hwang PH. The impact of diabetes mellitus on outcomes of endoscopic sinus surgery: A nested case-control study. *International Forum of Allergy and Rhinology* 2015; **5**:533-540.

15. Al Hilli MM, Bakkum-Gamez JN, Mariani A et al. The effect of diabetes and metformin on clinical outcomes is negligible in risk-adjusted endometrial cancer cohorts. *Gynecologic Oncology* 2016; **140**:270-276.

16. Ren B, Zhang Z-S, Liu W-W et al. Surgical outcomes following encephaloduroarteriosynangiosis in adult moyamoya disease associated with Type 2 diabetes. *Journal of Neurosurgery* 2016; **125**:308-314.

17. Choi Y, Choi Y, Choi CS, Lee Y-H. Diabetes mellitus increases the risk of intrahepatic recurrence of hepatocellular carcinoma after surgical resection. *Tumori* 2017; **103**:279-285.

18. Enomoto LM, Shrestha DP, Rosenthal MB, Hollenbeak CS, Gabbay RA. Risk factors associated with 30-day readmission and length of stay in patients with type 2 diabetes. *Journal of Diabetes and Its Complications* 2017; **31**:122-127.

19. Lopez-de-Andres A, Hernandez-Barrera V, Martinez-Huedo MA, Villanueva-Martinez M, Jimenez-Trujillo I, Jimenez-Garcia R. Type 2 diabetes and in-hospital complications after revision of total hip and knee arthroplasty. *Plos One* 2017; **12**.

20. Lanzetti RM, Lupariello D, Venditto T et al. The role of diabetes mellitus and BMI in the surgical treatment of ankle fractures. *Diabetes-Metabolism Research and Reviews* 2018; **34**.

21. Cakmak S, Canda AE, Ener K, Atmaca AF, Altinova S, Balbay MD. Does Type 2 Diabetes Mellitus Have an Impact on Postoperative Early, Mid-Term and Late-Term Urinary Continence After Robot-Assisted Radical Prostatectomy? *Journal of Endourology* 2019; **33**:201-206.

22. Lopez-de-Andres A, Perez-Farinos N, de Miguel-Diez J et al. Type 2 diabetes and postoperative pneumonia: An observational, population-based study using the Spanish Hospital Discharge Database, 2001-2015. *Plos One* 2019; **14**.

23. Creta A, Providencia R, Adragao P et al. Impact of Type-2 Diabetes Mellitus on the Outcomes of Catheter Ablation of Atrial Fibrillation (European Observational Multicentre Study). *American Journal of Cardiology* 2020; **125**:901-906.

24. Schootman M, Jeffe DB, Ratnapradipa KL, Eberth JM, Davidson NO. Increased 30-day mortality risk in patients with diabetes mellitus after colon cancer surgery: A mediation analysis. *Diseases of the Colon and Rectum* 2020:290-299.

25. Taimour S, Franzen S, Zarrouk M et al. Nationwide comparison of long-term survival and cardiovascular morbidity after acute aortic aneurysm repair in patients with and without type 2 diabetes. *Journal of Vascular Surgery* 2020; **71**:30-+.

26. Kodama T, Hayasaka S, Setogawa T. Plasma glucose levels, postoperative complications, and progression of retinopathy in diabetic patients undergoing intraocular lens implantation. *Graefe's Archive for Clinical and Experimental Ophthalmology* 1993; **231**:439-443.

27. Serna F, Mont MA, Krackow KA, Hungerford DS. Total knee arthroplasty in diabetic patients: Comparison to a matched control group. *Journal of Arthroplasty* 1994; **9**:375-379.

28. Blotter RH, Connolly E, Wasan A, Chapman MW. Acute complications in the operative treatment of isolated ankle fractures in patients with diabetes mellitus. *Foot and Ankle International* 1999; **20**:687-694.

29. Glassman SD, Alegre G, Carreon L, Dimar JR, Johnson JR. Perioperative complications of lumbar instrumentation and fusion in patients with diabetes mellitus. *Spine Journal* 2003; **3**:496-501.

30. Wiggans MG, Lordan JT, Shahtahmassebi G, Aroori S, Bowles MJ, Stell DA. The Interaction between Diabetes, Body Mass Index, Hepatic Steatosis, and Risk of Liver Resection: Insulin Dependent Diabetes Is the Greatest Risk for Major Complications. *HPB surgery : a world journal of hepatic, pancreatic and biliary surgery* 2014; **2014**:586159.

31. Verlato G, Marrelli D, Accordini S et al. Short-term and long-term risk factors in gastric cancer. *World journal of gastroenterology* 2015; **21**:6434-6443.

32. Najafian A, Selvarajah S, Schneider EB et al. Thirty-day readmission after lower extremity bypass in diabetic patients. *Journal of Surgical Research* 2016; **200**:356-364.

33. Qin C, Kim JYS, Hsu WK. Impact of insulin dependence on lumbar surgery outcomes. *Spine* 2016; **41**:E687-E693.

34. Ali TZ, Lehman EB, Aziz F. Unplanned return to operating room after lower extremity endovascular intervention is an independent predictor for hospital readmission. *Journal of Vascular Surgery* 2017; **65**:1735-1744.

35. Goltsman D, Morrison KA, Ascherman JA. Defining the Association between Diabetes and Plastic Surgery Outcomes: An Analysis of Nearly 40,000 Patients. *Plastic and reconstructive surgery Global open* 2017; **5**:e1461.

36. Worley N, Buza J, Jalai CM et al. Diabetes as an Independent Predictor for Extended Length of Hospital Stay and Increased Adverse Post-Operative Events in Patients Treated Surgically for Cervical Spondylotic Myelopathy. *International journal of spine surgery* 2017; **11**:10.

37. Haddix KP, Clement RC, Tennant JN, Ostrum RF. Complications Following Operatively Treated Ankle Fractures in Insulin- and Non-Insulin-Dependent Diabetic Patients. *Foot & ankle specialist* 2018; **11**:206-216.

38. Corrigan KE, Vargas MV, Robinson HN et al. Impact of Diabetes Mellitus on Postoperative Complications Following Laparoscopic Hysterectomy for Benign Indications. *Gynecologic and Obstetric Investigation* 2019; **84**:583-590.

39. Lung BE, Bisogno M, Kanjiya S, Komatsu DE, Wang ED. Early postoperative complications and discharge time in diabetic patients undergoing total shoulder arthroplasty. *Journal of orthopaedic surgery and research* 2019; **14**:9.

40. Power DJ, Sambrook PJ, Goss AN. The healing of dental extraction sockets in insulin-dependent diabetic patients: a prospective controlled observational study. *Australian dental journal* 2019; **64**:111-116.

41. Godshaw BM, Warren MS, Nammour MA, Chimento GF, Mohammed AE, Waddell BS. Insulin-Dependent Diabetic Patients are at Increased Risk of Postoperative Hyperglycemia When Undergoing Total Joint Arthroplasty. *Journal of Arthroplasty* 2020; **35**:2375-2379.

42. Jeger RV, Seeberger MD, Keller U, Pfisterer ME, Filipovic M. Oral hypoglycemics: Increased postoperative mortality in coronary risk patients. *Cardiology* 2007; **107**:296-301.

43. Ting CT, Chen RC, Chen CC, Liu MH, Chu D, Kuo NW. Diabetes worsens the surgical outcomes in cirrhotic patients with hepatocellular carcinoma. *Tohoku Journal of Experimental Medicine* 2012; **227**:73-81.

44. Tomita Y, Iwadoh K, Kutsunai K, Koyama I, Nakajima I, Fuchinoue S. Negative impact of underlying non-insulin-dependent diabetes mellitus nephropathy on long-term allograft survival in kidney transplantation: a 10-year analysis from a single center. *Transplant Proc* 2014; **46**:3438-3442.

45. Jørgensen CC, Madsbad S, Kehlet H. Postoperative morbidity and mortality in type-2 diabetics after fast-track primary total hip and knee arthroplasty. *Anesthesia and Analgesia* 2015; **120**:230-238.

46. Gebauer B, Meyer F, Ptok H et al. How does Diabetes Mellitus Influence Early Postoperative Results and Survival in Rectal Cancer? *Deutsche Medizinische Wochenschrift* 2018; **143**:e25-e33.

47. Baechle JJ, Smith PM, Bailey CE et al. Insulin-dependence predicts disease-specific survival in pancreatic neuroendocrine tumor patients. *HPB* 2019; **21**:S57.

48. Dolla C, Naso E, Mella A et al. Impact of type 2 diabetes mellitus on kidney transplant rates and clinical outcomes among waitlisted candidates in a single center European experience. *Scientific Reports* 2020; **10**.

49. Lee TC, Lee YL, Chen JC, Chen CH, Ho PS. Impact of type 2 diabetes on postoperative outcome after hip fracture: Nationwide population-based study in Taiwan. *BMJ Open Diabetes Research and Care* 2020; **8**.

50. Ortved M, Petersen PB, Jørgensen CC, Kehlet H. Postoperative Morbidity and Mortality in Diabetic Patients After Fast-Track Hip and Knee Arthroplasty: A Prospective Follow-up Cohort of 36,762 Procedures. *Anesthesia and analgesia* 2020.

51. Karamanos E, Sivrikoz E, Beale E, Chan L, Inaba K, Demetriades D. Effect of diabetes on outcomes in patients undergoing emergent cholecystectomy for acute cholecystitis. *World journal of surgery* 2013; **37**:2257-2264.

52. Lovecchio F, Beal M, Kwasny M, Manning D. Do patients with insulin-dependent and noninsulin-dependent diabetes have different risks for complications after arthroplasty? *Clin Orthop Relat Res* 2014; **472**:3570-3575.

53. Swaminathan S, Kakuturu J, Shetty S. Impact of Diabetes Mellitus on Bariatric Surgery Outcomes: A Metabolic and Bariatric Surgery Accreditation and Quality Improvement Program Analysis. *Journal of the American College of Surgeons* 2019; **229**:S23.

54. Lee D, Lee R, Gowda NB et al. Impact of diabetes mellitus on surgical complications in patients undergoing revision total knee arthroplasty: Insulin dependence makes a difference. *Journal of Clinical Orthopaedics and Trauma* 2020; **11**:140-146.

55. Bolsin SNC, Raineri F, Lo SK, Cattigan C, Arblaster R, Colson M. Cardiac complications and mortality rates in diabetic patients following non-cardiac surgery in an Australian teaching hospital. *Anaesthesia and Intensive Care* 2009; **37**:561-567.

56. Dosluoglu HH, Lall P, Nader ND, Harris LM, Dryjski ML. Insulin use is associated with poor limb salvage and survival in diabetic patients with chronic limb ischemia. *Journal of Vascular Surgery* 2010; **51**:1178-1189.

57. Hanninen M, McAlister FA, Bakal JA, van Diepen S, Ezekowitz JA. Neither Diabetes nor Glucose-Lowering Drugs Are Associated With Mortality After Noncardiac Surgery in Patients With Coronary Artery Disease or Heart Failure. *Canadian Journal of Cardiology* 2013; **29**:423-428.

58. Traven SA, Reeves RA, Walton ZJ, Woolf SK, Slone HS. Insulin-Dependence Predicts Surgical Complications and Hospital Admission Following Knee Arthroscopy. *The journal of knee surgery* 2020.
