# Supplementary material for: Postoperative short-term mortality between insulin-treated and non-insulin-treated patients with diabetes after non-cardiac surgery: a systematic review and meta-analysis
Source: Front Med (Lausanne). 2023 May 2;10:1142490. doi: 10.3389/fmed.2023.1142490 (PMC10185903; doi:10.3389/fmed.2023.1142490)
Supplement: Supplementary file 1 [file Data_Sheet_1.zip › Supplemental Digital Content 3revised.docx]

Supplemental Digital Content 3

Postoperative short-term mortality between insulin-treated and non-insulin-treated patients with diabetes after non-cardiac surgery: a systematic review and meta-analysis

## Table A. 1. GRADE summary of findings.

| **Quality assessment** | | | | | | | **No of patients** | **Effect** | **Quality** | **Importance** |  |
| --- | --- | --- | --- | --- | --- | --- | --- | --- | --- | --- | --- |
|  |  |  |  |  |  |  |  |  |  |  |  |
| **No of studies** | **Design** | **Risk of bias** | **Inconsistency** | **Indirectness** | **Imprecision** | **Other considerations** | **Total** | **Risk ratio (95% CI)** |  |  |  |
| **30-day mortality** | | | | | | | | | | |  |
| 19 | observational studies | serious^1^ | serious^2^ | no serious | no serious^3^ | potential reporting bias^4^ | 197704 | 1.305 (1.127 to 1.511)  - | ⊕OOO VERY LOW | CRITICAL |  |
| **Hospital mortality** | | | | | | | | | | |  |
| 2 | observational studies | serious^5^ | no serious | no serious | serious^6^ | potential reporting bias^4^ | 9032 | 0.970 (0.584 to 1.611)  - | ⊕OOO VERY LOW | CRITICAL |  |

^1^ The NOS score of the studies is 4 to 6 points
^2^ Subgroup analysis results were inconsistent with pooled results. Subgroup-analysis was plausible, but overall judged unlikely. Present pooled estimate, rate down for insistency
^3^ Total sample size was larger than the calculated optimal information size (OIS) (197704 vs 61409, α= 0.05, β= 0.20, relative risk was assumed 1.6335, control event rate was 0.0027, the median of the available studies). The 95% confidence interval for RR does not include 1.0.
^4^ Potential publication bias existed. Downgraded by one level.

^5^ The NOS score of the studies is 4 points
^6^ Total sample size was lowerr than the calculated optimal information size (OIS) (9032 vs 61409, α= 0.05, β= 0.20, relative risk was assumed 1.6335, control event rate was 0.0027, the median of the available studies). The 95% confidence interval for RR does not include 1.0.
^7^ Only two studies were included. Potential publication bias existed. Downgraded by one level.
