# Supplementary material for: Postoperative short-term mortality between insulin-treated and non-insulin-treated patients with diabetes after non-cardiac surgery: a systematic review and meta-analysis
Source: Front Med (Lausanne). 2023 May 2;10:1142490. doi: 10.3389/fmed.2023.1142490 (PMC10185903; doi:10.3389/fmed.2023.1142490)

Supplemental Digital Content 4

Postoperative short-term mortality between insulin-treated and non-insulin-treated patients with diabetes after non-cardiac surgery: a systematic review and meta-analysis

## Figure 1. Sensitivity analysis


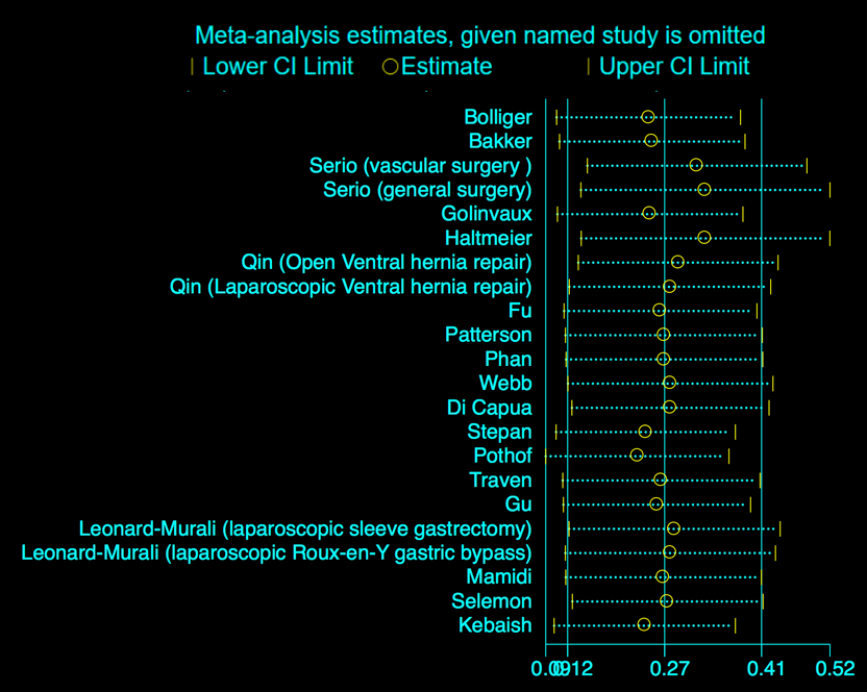

Supplement: Supplementary file 1 [file Data_Sheet_1.zip › Supplemental Digital Content 4revised.docx]
